# A description of a tailored knowledge translation intervention delivered by knowledge brokers within public health departments in Canada

**DOI:** 10.1186/s12961-019-0460-z

**Published:** 2019-06-20

**Authors:** Maureen Dobbins, Lori Greco, Jennifer Yost, Robyn Traynor, Kara Decorby-Watson, Reza Yousefi-Nooraie

**Affiliations:** 1 Faculty of Health Sciences, School of Nursing, McMaster University, 175 Longwood Road South, Suite 210A, Hamilton, Ontario Canada; 2 Region of Peel - Public Health, 7120 Hurontario Street, Mississauga, Ontario Canada; 3 M. Louise Fitzpatrick College of Nursing, Villanova University, Driscoll Hall, Room 330, 800 Lancaster Avenue, Villanova, PA United States of America; 4 Department of Community Health and Epidemiology, Dalhousie University, Halifax, Nova Scotia Canada; 5 Centre for Person-Centered Practice Research, Queen Margaret University Edinburgh and Affiliate Member, Queen Margaret University, Edinburgh, United Kingdom; 6 Department of Public Health Sciences, University of Rochester, School of Medicine, 265 Crittenden Blvd., CU 420644, Rochester, New York 14642 United States of America

**Keywords:** Evidence-informed decision-making, public health, knowledge translation, capacity-building, knowledge broker

## Abstract

**Background:**

While there is an expectation to demonstrate evidence-informed public health there is an ongoing need for capacity development. The purpose of this paper is to provide a description of a tailored knowledge translation intervention implemented by knowledge brokers (KBs), and reflections on the factors that facilitated or hindered its implementation.

**Methods:**

The 22-month knowledge translation intervention, implemented by two KBs, sought to facilitate evidence-informed public health decision-making. Data on outcomes were collected using a knowledge, skills and behavioural assessment survey. In addition, the KBs maintained reflective journals noting which activities appeared successful or not, as well as factors related to the individual or the organisation that facilitated or hindered evidence-informed decision-making.

**Results:**

Tailoring of the knowledge translation intervention to address the needs, preferences and structure of each organisation resulted in three unique interventions being implemented. A consistent finding across organisations was that each site needed to determine where evidence-informed decision-making ‘fit’ within pre-existing organisational processes. Components of the intervention consistent across the three organisations included one-to-one mentoring of teams through rapid evidence reviews, large group workshops and regular meetings with senior management. Components that varied included the frequency of the KB being physically onsite, the amount of time staff spent with the KB and proportion of time spent one-to-one with a KB versus in workshops. Key facilitating factors for implementation included strong leadership, influential power of champions, supportive infrastructure, committed resources and staff enthusiasm.

**Conclusions:**

The results of this study illustrate the importance of working collaboratively with organisations to tailor knowledge translation interventions to best meet unique needs, preferences, organisational structures and contexts. Organisational factors such as leadership, champions and supportive infrastructure play a key role in determining the impact of the knowledge translation interventions. Future studies should explore how these factors can be fostered and/or developed within organisations. While KBs implemented the knowledge translation intervention in this study, more research is needed to understand the impact of all change agent roles including KBs, as well as how these roles can be maintained in the long-term if proven effective.

## Background

Public health professionals in Canada are expected to deliver effective and efficient programmes and services that protect and promote the health of the population [1]. Evidence-informed decision-making (EIDM) is one component of public health decision-making that can support the implementation of effective and efficient programmes and services. EIDM is defined as the incorporation of the best available research evidence along with knowledge related to the setting, political and societal preferences, resources and public health expertise into decisions [2, 3]. The National Collaborating Centre for Methods and Tools (NCCMT) advocates that EIDM is comprised of seven steps, namely ‘define’ (a searchable question), ‘search’ (for evidence), ‘appraise’ (the quality of evidence), ‘synthesise’ (the evidence), ‘adapt’ (to the local context), ‘implement’ (a change in practice) and ‘evaluate’ (impact) [4]. Research has shown EIDM is associated with more effective policies and programmes being implemented, as well as optimal use of scarce public health resources [2, 5].

While the public health sector in Canada has been working toward EIDM for many years [6], knowledge translation (KT) interventions are still needed to support public health organisations in developing the culture, context and infrastructure to support EIDM, as well as to assist public health professionals in obtaining the knowledge, skills and capacity necessary to practice in an evidence-informed way [7, 8]. Research has shown that early decision-maker involvement in the research process, messages tailored to decision-maker needs and attention to context help facilitate EIDM [9]. Research also suggests that activities that address both context and individual capacity hold promise for achieving EIDM [10, 11]. While many KT interventions have been evaluated, questions still remain as to how best to support EIDM in public health.

Knowledge brokers (KB) are one type of change agent that may be used to implement KT interventions. KBs work to promote, facilitate and support EIDM through one-to-one interaction with individuals, teams and organisations [12, 13]. One way in which KBs function is to provide a link between researchers and practitioners, decision-makers and policy-makers [14], whereas another is to link knowledge users to the scientific literature while supporting the development of capacity for EIDM [12, 15]. KBs assess the need for organisational change, develop strategies to facilitate change and work to create a culture that values the use of the best available evidence in decision-making [9, 16]. They are instrumental in facilitating communication and knowledge sharing among key stakeholders [17], facilitating learning [18–21], building capacity to locate, appraise and translate evidence into the local context [18, 22, 23], and improving interpretation of research findings and development of implications for action [24]. While building individual knowledge, skills and capacity for EIDM, KBs also support the development of organisational context and infrastructure that supports emerging EIDM behaviours among staff [12, 25]. It is believed that KBs must possess exceptional interpersonal, communication and motivational skills, as well as expertise from both the knowledge user’s perspective and that of the research community to be effective [26–28].

In partnership with three Canadian public health departments, we used a case study design to conduct this study. The purpose of this study was to enhance individual capacity for EIDM and facilitate organisational contexts conducive towards it. Through the use of a case study methodology, we evaluated the impact of a KT intervention on EIDM knowledge, skills and behaviour, and identified contextual factors that facilitated or impeded impact. In this paper, we provide a thorough description of the KT intervention implemented by KBs in the three public health departments. Details related to the activities that were conducted and how they were implemented are provided such that others may replicate the intervention in other settings. The level of detail goes beyond what is generally included in publications reporting intervention outcomes. However, greater knowledge of what the intervention was comprised of and issues specifically related to implementation are needed in the KT literature. The primary focus of this paper is therefore to discuss important aspects related to intervention implementation and reflect on key factors that either facilitated or hindered its implementation.

## Methods

### Participants

This case study was funded by the Canadian Institutes of Health Research as part of the Partnerships for Health System Improvement competition (FRN101867). Participants included front-line public health practitioners, managers, directors, and Medical and Associate Medical Officers of Health from three public health departments (cases). Ethics approval was received from the McMaster University Research Ethics Board.

### Setting

The three cases were situated in southwestern Ontario and differed with respect to the number of full-time staff, size of population served and organisational structure. They all served both urban and rural areas, as well as ethnically diverse populations, and were organised in discrete divisions or directorates such as communicable disease, chronic disease prevention and environmental health. Additional details related to the three cases are presented in Dobbins et al. 2018 [29].

### KT intervention

The KT intervention targeted six of the seven steps in the evidence-informed public health process described by the NCCMT [30]. This included identifying a practice-based question, developing and conducting a search for relevant evidence, appraising the methodological quality of the retrieved evidence, synthesising the evidence, assessing the applicability of the evidence to the local context, and determining if changes to local public health policy and practice were necessary as a result of reviewing the evidence. A KB implemented the KT intervention in each case through large and small group interactive workshops, one-to-one mentoring through the process of conducting a rapid evidence review via face-to-face, electronic and telephone communication, attending meetings and presentations with senior management, and assisting in the development of policies and procedures that supported EIDM. The research team worked with each case to determine what form of interaction was preferred between the KB and staff (i.e. amount of time and in what manner – one-to-one versus small or large-group training).

The intervention was tailored for each case taking into account the philosophical stance, existing processes and supports, EIDM knowledge and skill of staff, and organisational goals. The intervention was implemented over 22 months. Each case was assigned a KB. While the KBs were employed by McMaster University, they spent a substantial amount of time located at the case sites. Given the time required to deliver the KT intervention, it was necessary to have two KBs, where one was assigned two of the cases and the other KB was assigned one case. Both KBs held a Masters degree and had several years’ experience working in KT and/or public health. In each case, the intervention began with an organisational assessment, followed by intervention implementation and ongoing monitoring and refinement of the intervention throughout the study. Additional details about the organisational assessment and results of this assessment have been published previously [29]. A more detailed description of the KT intervention implemented in each case is provided in Table 1.

**Table 1:** Details of the knowledge translation intervention, tailored for each health department

	Case A	Case B	Case C
Context	Large, diverse population served; Medical Officer of Health had vision for EIDM; EIDM a strategic priority; committed resources	Large, urban centre served; Medical Officer of Health strongly committed to EIDM; manager ‘champion’ for EIDM; EIDM a strategic priority	Mid-size, urban/rural mix served; Medical Officer of Health committed to EIDM; commitment from Executive team
Intervention	September 2010 – June 2012	April 2011 – February 2013	April 2011 – December 2012
KB on-site vs. virtual	100% on-site (2 days/week onsite for 22 months)	25% on-site; 75% virtual (2 days/month onsite for first 6 months, then ½ day/week for 16 months)	25% on-site; 75% virtual (2 days/month onsite for first 6 months, then 1 day every other week for 16 months)
Organisational-level strategies	Workshop training for all staff; and KB participated in intra-department presentations	Introductory workshops provided to consultants; EIDM training for all staff in one directorate	Department-wide EIDM training through large group workshops; KB advised Research and Knowledge Exchange Committee on creation of an EIDM guidebook; development of an EIDM organisational policy
Group-level strategies	KB mentored staff teams through rapid evidence reviews and provided EIDM training		
Individual-level strategies	KB mentored individual staff and was available for ad-hoc EIDM-related questions		
Senior Management interaction	Regular meetings and presentations to Senior Management Team	KB advocated for staff time to be allocated to EIDM and advised Senior Management Team	KB advised Executive team on EIDM policy and procedures
Total activities	18 rapid evidence reviews, large-scale training sessions, KB facilitated/contributed to Critical Appraisal Club and presentations of research to staff colleagues and Senior Management	5 rapid evidence reviews; additional divisional training delivered; abstracts submitted to present research; presentations to Senior Management	5 rapid evidence reviews; EIDM policy and procedures developed and approved; presentations to Executive team; all-staff training delivered

*EIDM* evidence-based decision-making, *KB* knowledge broker

#### Ongoing monitoring and refinement of the KT intervention

The KBs, research team and senior management met regularly throughout the study to discuss intervention implementation and progress. During these discussions, ideas about how to continually modify the intervention arose and, in most instances, they were implemented. Case A identified new tools it wanted to use to support specific steps in the rapid evidence review process and revised existing tools. At the organisation level, Case A identified that additional strategies were needed to clarify staff roles and responsibilities with respect to EIDM. The KB also worked on developing workshop content and delivering EIDM training for all staff.

In Case B, an ‘EIDM roadmap’ was developed to help support and sustain EIDM. Identified responsibilities included developing and implementing brokering strategies, recommending communication within or across directorates to help avoid duplication of rapid evidence reviews or supporting work, clarifying roles in EIDM broadly and for KB work specifically, helping the health department negotiate a selection of priority questions, and generating practical recommendations for internal consultants.

Case C implemented an EIDM policy that was ultimately endorsed and adopted by the health department’s Executive Committee. With approval from senior management, the purpose of an existing committee was revised to include responsibility for developing and implementing strategies to sustain EIDM in the organisation. This health department also implemented voluntary basic EIDM training for all employees comprised of a full day EIDM introductory workshop and a subsequent half day workshop on critical appraisal of systematic reviews.

#### Evaluation method and measures

An estimate of intervention dose (i.e. overall time and type) and intensity according to level (i.e. organisation, group, individual) was tracked by the KBs in a reflective journal. KBs maintained a daily reflective journal of activities undertaken, as well as observations and reflections of the impact of these activities on EIDM. KBs also documented key factors that either facilitated or hindered implementation. Data from the KBs’ reflective journals were entered in NVIVO 8. The data were coded for emerging trends and themes by several members of the research team (RT, LG, KD, MD). Once agreement on major themes and codes were reached, and a coding scheme developed, the data were coded by one member of the research team (RT). The research team met to discuss interpretation of the themes.

## Results

The KBs spent 2 days per week delivering the KT intervention in each case, although there were substantial differences in how the time was allocated. In Case A, the KB was physically onsite 2 days per week for the full 22 months. The KB spent the majority of time mentoring teams through rapid evidence reviews, and assessing the applicability of the research evidence to the local setting. The KB also participated in a monthly critical appraisal club with staff, provided workshops on EIDM, responded to questions posed by staff related to EIDM, and attended and presented at meetings with senior management and other health department staff. Presentations included an introduction to EIDM, interpreting statistics, and searching for and critically appraising public health practice guidelines. The total number of staff who were mentored, either individually or in small groups by the KB, was 48, whereas 33 staff participated in large group training such as presentations or workshops*.*

In Case B, the KB was initially physically onsite 2 days during 1 week of the month, but after approximately 6 months, at the request of the organisation, this was changed to half a day each week. The organisation indicated having the KB onsite more frequently would help maintain momentum for those engaged with the KB. The KB spent time onsite engaged in two specific activities, namely (1) building capacity for EIDM among staff who had responsibility for incorporating research evidence into practice decisions; and (2) conducting workshops with other staff about what EIDM is and why it is important. The KB also attended and presented at meetings with the senior management team and other health department staff. Twelve staff were mentored either individually or in small groups, by the KB; 76 participated in large group training such as presentations or workshops.

In Case C, the KB also started out being physically onsite 2 days during 1 week of the month, but 6 months into the intervention, the case requested this be changed to 1 day every other week. The KB spent more time conducting large group training workshops, although small groups were also mentored through rapid evidence reviews. The KB also worked with the Research and Knowledge Exchange committee to develop policies, procedures and a guidebook for EIDM, and presented at senior management meetings and department-wide symposia. The total number of staff that engaged either individually or in small groups with the KB was 17, whereas 49 participated in large group training such as presentations or workshops.

Overall, a statistically significant improvement in knowledge and skill was observed from baseline to follow-up in each case. Further details and discussion of these results have been reported elsewhere [29]. The remainder of this paper focuses on the results of observations from implementing the intervention through assessment of the KBs’ reflective journals.

### Implementation facilitators and challenges

The following are the key factors – at the organisational, team and individual level – perceived by the KBs that either facilitated or hindered implementation of the KT intervention and, ultimately, EIDM.

#### Facilitators

Particularly important factors in supporting EIDM at the organisational level were strong leadership and support for changing culture and practice to systematically incorporate research evidence into the decision-making process. Champions to communicate the value of EIDM supported ‘getting the word out’ and increased knowledge and understanding among staff about what EIDM is and is not. Existing infrastructure, such as a library and on-staff librarians, was identified as a key factor that enabled EIDM, as well as having particular roles identified in the organisation with responsibility for finding and using research evidence. Each case recognised the existence of committed financial and human resources, willingness to participate in the research study and support for staff to spend time engaged in the EIDM process as fundamental to the EIDM process. Existing EIDM processes and tools, such as those from the NCCMT and Health Evidence, helped the cases adopt or develop their own EIDM policies, procedures and tools. An important component of the KT interventions, observed by the KBs as contributing to successful EIDM, was regular contact between the KBs, and KBs and staff, research team, case key contact, and senior management.

At the team level, the KBs identified meaningful interest in EIDM among teams and enthusiasm to improve knowledge and skills in finding and using research evidence in decision-making as important factors. This was observed especially among teams that were working on resolving important priority policy or programme decisions in their health department. The KBs noted that small teams working on rapid evidence reviews on topics of relevance and importance to the health department were seen among their peers as ‘taking small steps’, which enabled the health department to pilot EIDM processes and ‘test’ whether EIDM was feasible and valuable for making important practice decisions. In some cases, teams that were already skilled in finding and using research but who wanted to formalise their processes, were instrumental in demonstrating the feasibility and usefulness of EIDM. Sharing early experiences reassured and provided support for teams who later engaged in EIDM.

At the individual level, the KBs noted that curiosity and enthusiasm among staff supported culture change and the creation of EIDM processes and practices. The KBs indicated that identifying staff with existing EIDM-related training and experience and getting them involved in the intervention was helpful, particularly in the early stages of implementing the KT intervention.

#### Challenges

The KBs observed that challenges to implementing the intervention mirrored facilitators to a large extent. For example, without leadership, champions, infrastructure and committed resources, the KBs noted that the cases struggled to achieve culture and practice changes that formally and systematically incorporated research evidence into decision-making. At the organisational level, KBs commented that limited engagement in and modelling of EIDM behaviours by senior management tended to result in similar behaviours by staff throughout the organisation. The KBs reflected that organisational change required active involvement of leadership that was consistently observable throughout the organisation. They also noted that verbal communication of the organisation’s commitment to EIDM was insufficient in facilitating organisational change.

At the team level, the KBs observed how managers could act as barriers to staff development with EIDM. In instances where managers lacked understanding of the EIDM process or the time required to develop skills for EIDM and conduct activities consistent with EIDM, KBs observed staff did not have adequate time allocated to these tasks. As a result, staff struggled to make progress and/or experienced pressure to complete their work more quickly. Further, when teams reported their public health work as ‘mandated’ or externally prescribed, it was challenging to engage staff in EIDM as they did not perceive opportunities existed to change practice. This significantly reduced interest and motivation to learn EIDM-related skills across some teams and divisions/directorates.

At the staff level, individuals varied greatly with respect to the amount of EIDM-related training they required to integrate EIDM in day-to-day work. This presented challenges with respect to developing multiple unique training programmes at the individual and team levels. Other challenges included occasional resistance to participation in EIDM and to changing practice status quo. A lack of confidence among staff regarding EIDM-related knowledge and skill was consistently identified. Most challenging was the reality of competing priorities and lack of time. This was sometimes characterised as staff not seeing EIDM as part of ‘core work’, but rather an ‘add-on’ to their already full workloads.

## Discussion

Each case involved in this study featured a unique combination of individual, team and organisational characteristics and circumstances relating to their communities, political contexts and available resources. Furthermore, they were involved, to varying extents, in supporting EIDM prior to this study and therefore may have been more amenable to EIDM adoption in comparison to other public health departments in Ontario and Canada. Each case was involved in designing its own KT intervention, which has been shown in other studies to be associated with a higher degree of uptake of research evidence [31].

As part of the KT intervention, KBs actively helped public health professionals identify, appraise and interpret best available research evidence as well as assess its applicability to the local context and determine if practice changes were needed based on review of the evidence. Previous evidence has shown that active engagement of decision-makers in applying evidence to practice improves uptake of research evidence [6].

The findings of this study are supported by others who have reported that KT interventions need to be tailored to the specific context of organisations and the health professionals working within them [6, 28]. Furthermore, the complexity of the KT intervention implemented in this study is also supported by others who have suggested that EIDM is much more complex than previously thought and that it is likely that single, one-off interventions such as workshops or journal clubs are insufficient to realise EIDM in health organisations, including public health [32, 33]. Current thinking supports an organisation-wide approach to EIDM [33].

A variety of change agents have been used to implement KT interventions, including facilitators [34, 35], opinion leaders [9, 36], academic detailers and KBs. KBs have been shown previously to promote and enhance EIDM [9] and to be adaptable to different contexts [22]. In this study of public health departments, the KT intervention implemented by KBs was associated with statistically significant improvements in EIDM knowledge, skills and behaviours [29]. Others have reported that KT interventions implemented by KBs were effective in facilitating and improving communication and knowledge sharing between key stakeholders [17], in building capacity to locate, appraise and translate evidence to the local context, and in increasing skill to interpret and apply research evidence to the practice setting [19, 21].

In this case study, particular activities were highly important at the outset of the intervention in setting the stage for intervention implementation; specifically, plans to draft a policy and procedures guidebook, with subsequent rapid evidence review experience that informed the tools for a guidebook, meetings with the Medical Officers of Health, and development of a document outlining a process for change at both the team and organisation levels. These activities acted as catalysts in each of the health departments. KBs have been shown previously to be catalysts for systems change [17], as well as establishing and nurturing connections within organisations [28]. Questions remain, however, with respect to optimal timing for intervention initiation and how to sustain EIDM in the organisation(s).

The KBs in this study supported participants to gain capacity in locating, appraising, translating and assessing applicability and applying evidence to practice, as well as assisting each case to improve its EIDM processes. The KBs identified librarians in two of the cases as champions for EIDM, while the third case identified library services as a barrier to EIDM [37]. Other factors identified as important to EIDM by the KBs included available resources, participation of the local community, cultural appropriateness, and social and political issues [38]. The KBs acted as mentors, gradually transferring responsibility to decision-makers as skills improved, eventually enabling staff to work with minimal supervision or support. Others have reported similar findings with respect to the KB role in implementing KT interventions as evolving over time as knowledge and skill among the workforce improves [22, 24].

The findings of this study demonstrate an increase in EIDM knowledge and skills among staff and improvement in EIDM behaviours, particularly among staff who interacted closely with the KBs. Findings also highlight the importance of infrastructure and policies aimed at maintaining and sustaining a culture of EIDM, which has also been reported in the literature [25]. Tangible structures and resources, which were identified in this study as playing a crucial role in EIDM, have also been reported by others to be an important precursor to organisational change [39]. The findings of this study suggest that tailored KT interventions implemented in public health departments are promising for encouraging and supporting EIDM. The findings of this study also suggest that the intervention can lead to impact when implemented by KBs in public health departments. However, questions remain as to how setting and context affect the impact of change agents on EIDM, despite the publication of multiple studies on this topic over the past 10 years. Additional research is needed to more fully understand the impact of these various change-agent roles, as well as concepts related to organisational readiness for change, supporting culture change, attaining leadership commitment to EIDM, and how setting and context affect change-agent impact.

## Conclusions

The results of this study illustrate the importance of working collaboratively with organisations to tailor KT interventions to best meet unique needs, preferences, organisational structures and contexts. Organisational factors, such as leadership, commitment to culture change, available resources and infrastructure, are critical in supporting the change process. Public health departments considering embarking on a process to embed EIDM into routine practice should first identify organisational strengths for EIDM, and work to optimise organisational supports where they exist and develop them where they do not. Once these are in place, organisations are more likely to be ready for change and to engage actively in developing and implementing KT strategies. While KBs implemented the KT intervention in this study, more research is needed to understand the impact of all change-agent roles, including KBs, as well as how these roles can be maintained in the long-term if proven effective.

## Data Availability

Data for this study are housed on a password-protected server at McMaster University, and available upon request from the primary author.

## Abbreviations

EIDM: evidence-informed decision-making
KB: knowledge broker
KT: knowledge translation
NCCMT: National Collaborating Centre for Methods and Tools
